# Epidermal Growth Factor, through Alleviating Oxidative Stress, Protect IPEC-J2 Cells from Lipopolysaccharides-Induced Apoptosis

**DOI:** 10.3390/ijms19030848

**Published:** 2018-03-12

**Authors:** Xiaopeng Tang, Bo Liu, Xiangrong Wang, Qifang Yu, Rejun Fang

**Affiliations:** 1 College of Animal Science and Technology, Hunan Agricultural University, Changsha 410128, China; tangxiaopeng110@126.com (X.T.); xb19940608@163.com (B.L.); cooliowxr@163.com (X.W.); yqqah@126.com (Q.Y.); 2 Hunan Co-Innovation Center of Animal Production Safety, Changsha 410128, China

**Keywords:** antioxidant, apoptosis, epidermal growth factor, lipopolysaccharides (LPS), oxidative stress

## Abstract

The epidermal growth factor (EGF) has been widely used for protection of stress-induced intestinal mucosa dysfunction. However, whether EGF would alleviate oxidative injury and reduce apoptosis in porcine intestine is not yet known. Therefore, the aim of this study was to investigate the effect of EGF on lipopolysaccharides (LPS)-induced induction of oxidative stress and ensuing apoptosis in the porcine intestinal epithelial cell line, IPEC-J2. The present study showed that EGF significantly increased cell viability and decreased the LPS-induced induction of apoptosis, dehydrogenase (LDH) release and malonaldehyde (MDA) production. EGF also (i) decreased expression of the pro-apoptotic genes *Fas*, *Bax*, *Cascase-3*, *Cascase-8*, *Cascase-9*, and proteins such as P53, Fas, Bax, Caspase3; (ii) increased antiapoptotic protein B-cell lymphoma 2 (Bcl2) expression; (iii) increased mRNA levels of the nuclear factor erythroid 2-related factor 2 (*Nrf2*) related genes Nrf2, manganese superoxide dismutase (*SOD2*), catalase (*CAT*), glutathione peroxidase (*GSH-Px*), heme oxygenase (*HO-1*) and quinone oxidoreductase (*NQO1*); (iv) protein level of Nrf2-realeted proteins Nrf2, HO-1, NQO1; and (v) total antioxidant capacity (T-AOC), CAT, SOD, GSH-Px concentrations. Collectively, our results indicated that EGF enhanced Nrf2 protein expression, and upregulated the expression of phase II metabolizing enzymes (such as HO-1 and NQO1) and antioxidative enzymes (SOD, CAT and GSH-Px) to alleviate oxidative injury, and then protect IPEC-J2 cells from apoptosis induced by LPS.

## 1. Introduction

The intestinal epithelium is formed by a continuous monolayer of proliferating and differentiating intestinal epithelial cells (IECs) which separate the intestinal mucosa from the lumen environment [1]. IECs, an effective semipermeable barrier that allows the absorption of nutrients and water, limits the permeation of toxins, allergens and pathogens from the gut lumen into mucosal tissue and circulation [2]. A strict balance between cellular proliferation and apoptosis is necessary to maintain the barrier function of IECs [3]. However, stressful conditions such as oxidative stress may impair the intestinal barrier [4], resulting in an increase in permeation of toxins and allergens. Oxidative stress, recognized as a state of imbalance between the production of reactive oxygen species (ROS) and antioxidant defenses [5,6], has been reported to induce cell apoptosis in both in vitro and in vivo experiments [4,7,8]. Normal cells have a variety of defense mechanisms including, but not limit to, non-enzymatic antioxidant systems (such as ascorbic acid, vitamin E, and glutathione) and enzymatic antioxidant systems (such as superoxide dismutase (SOD), catalase (CAT), and Glutathione peroxidase (GSH-Px)) [9,10]. LPS, a major integral component of the outer membrane of Gram-negative bacteria, has been shown to increase oxidative injury in IECs by producing numerous ROS, which can lead to lipid peroxidation and apoptosis [11,12,13,14]. The present study used LPS to establish a cell injury model to study the protective effects of EGF on IPEC-J2 cells.

EGF is a cytoprotective peptide consisting of 53 amino acid residues, which plays important roles in the homeostasis of intestinal mucosa maintenance [1,15,16]. Previous studies have demonstrated that EGF provided protection against alcohol-induced inflammation [17], hydrogen peroxide [18] and acetaldehyde [19], induced barrier dysfunction of epithelium cells, and ischemia/reperfusion-induced oxidative injury [20]. However, little is known as to whether EGF would alleviate oxidative injury and reduce apoptosis in porcine intestine. We hypothesize that EGF attenuates the LPS-induced oxidative injury, thus, blocking apoptosis of IPEC-J2 cells. To test this hypothesis, LPS was used to establish the cellular injury model to examine the protective effects of EGF on LPS-induced oxidative injury and apoptosis of IPEC-J2 cells.

## 2. Results

### 2.1. Effects of EGF on the Growth of IPEC-J2 Cells

**Figure 1:**
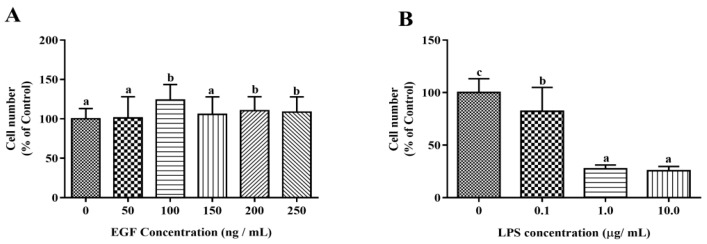

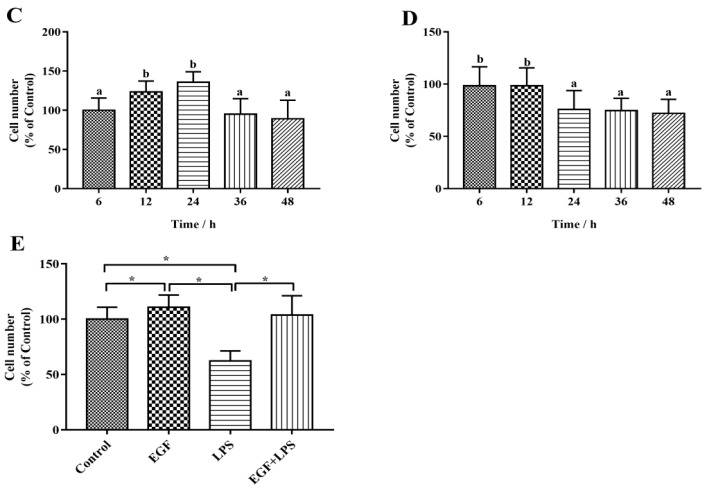
Effects of EGF on cell growth. (**A**) Toxicity of EGF on cell growth; (**B**) Toxicity of LPS on cell growth; (**C**) Time-dependent effects of EGF on cell growth; (**D**) Time-dependent effects of LPS on cell growth; (**E**) Effects of EGF on IPEC-J2 cells growth challenged by LPS. Data are expressed as mean ± SD, *n* = 6, values with different letters (a, b, c) are significantly different (*p* < 0.05), * *p* < 0.05.

The cytotoxic effects of EGF and LPS on IPEC-J2 cells were evaluated by CCK-8 assay. The growth of IPEC-J2 cells is shown in Figure 1. The results showed that EGF at 100 ng/mL significantly increased (*p* < 0.05) the cell numbers compared to 0, 50, 150 ng/mL EGF group, and no difference to control was found with to 200, 250 ng/mL EGF group (Figure 1A); LPS at 1.0 μg/mL caused a significant reduction (*p* < 0.05) of IPEC-J2 cell numbers (Figure 1B). IPEC-J2 treated with 100 ng/mL EGF for 24 h had a higher (*p* < 0.05) cell growth rate (Figure 1C); IPEC-J2 cells treated with1.0 μg/mL LPS for 24, 36, 48 h, the cell numbers reduced significant (*p* < 0.05) (Figure 1D). Accordingly, 100 ng/mL EGF, 1.0 μg/mL LPS, and cell cultured for 24 h were chosen for subsequent experiments. To explore the protective effect of EGF on cell viability, IPEC-J2 cells were treated with 100 ng/mL EGF and/or 1.0 μg/mL LPS for 24 h, as shown in Figure 1E, EGF significantly (*p* < 0.05) increased cell growth challenged by LPS.

### 2.2. EGF Reduces LDH and MDA Production Induced by LPS in IPEC-J2 Cells

**Figure 2:**
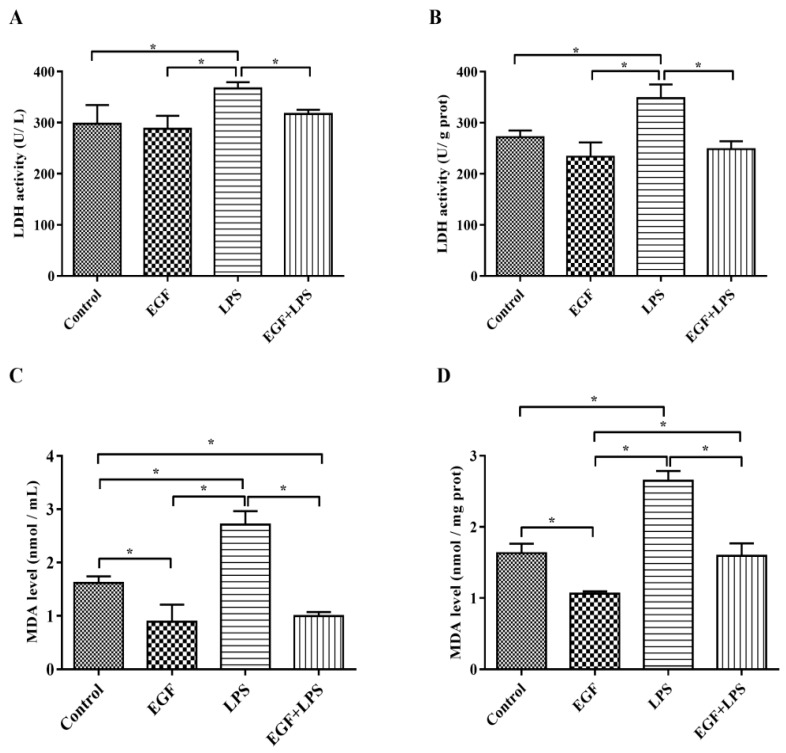
Effects of EGF on LDH and MDA production. (**A**) LDH release into medium; (**B**) LDH level in cells; (**C**) MDA content in medium; (**D**) MDA content in cells. Data are expressed as mean ± SD, *n* = 6, * *p* < 0.05.

To further explore the protective effect of EGF, the production of LDH and MDA, the indicators of cell injury, were also examined in LPS-challenged IPEC-J2 cells. The results showed that the amounts of LDH released into the medium (Figure 2A) and in cells (Figure 2B) were higher (*p* < 0.05) in IPEC-J2 cells treated with LPS, and the levels of MDA in medium (Figure 2C) and in cells (Figure 2D) were also higher (*p* < 0.05) in IPEC-J2 cells treated with LPS, which indicated that the cell membrane integrity was affected. EGF decreased LDH release and MDA production significantly (*p* < 0.05) in IPEC-J2 cells challenged by LPS, affirmed that EGF had a protective effect on IPEC-J2 cells oxidative injury.

### 2.3. EGF Increased Antioxidant Enzyme Secretion in IPEC-J2 Cells

**Figure 3:**
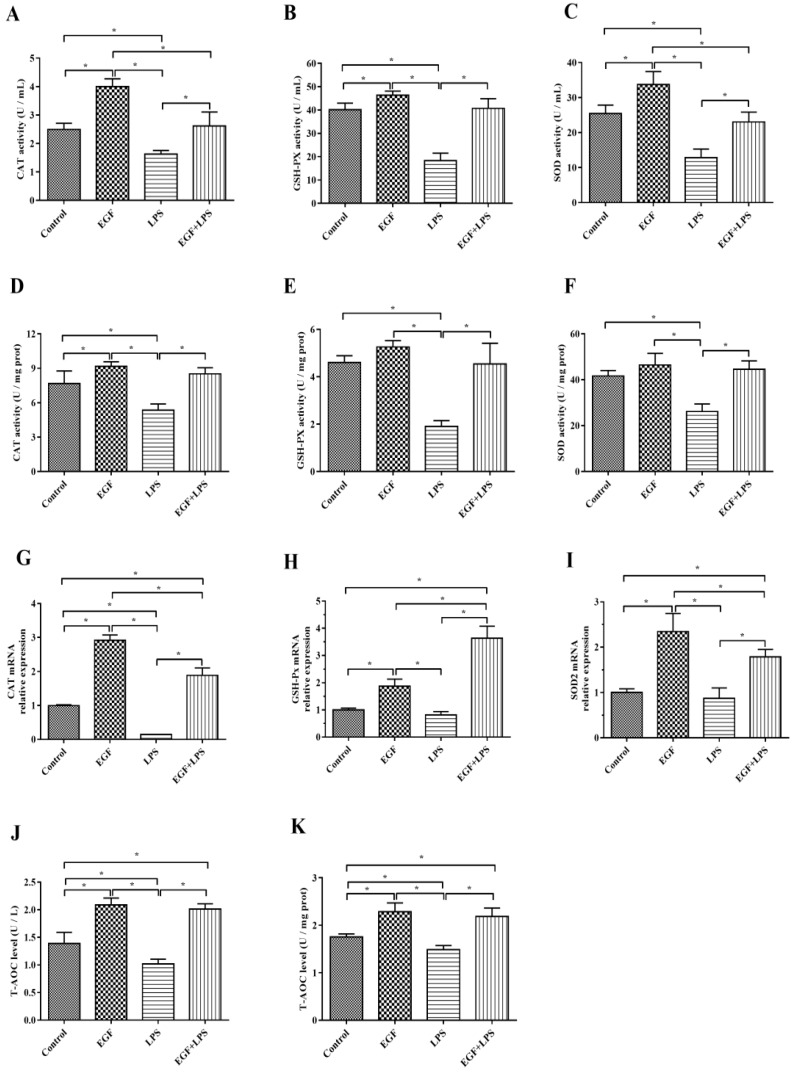
Effects of EGF on antioxidation capacity of IPEC-J2 cells challenged by LPS. (**A**) CAT activity in cell supernatant; (**B**) GSH-PX activity in cell supernatant; (**C**) SOD activity in cell supernatant; (**D**) CAT activity in cells; (**E**) GSH-PX activity in cells; (**F**) SOD activity in cells; (**G**) *CAT* gene expression; (**H**) *GSH-PX* gene expression; (**I**) *SOD2* gene expression; (**J**) T-AOC levels in cell supernatant; (**K**) T-AOC levels in cells. Data are presented as mean ± SD, *n* = 3, * *p* < 0.05.

The results showed that in IPEC-J2 cells challenged with LPS, T-AOC, CAT, GSH-Px and SOD in cells and supernatants were significantly reduced (*p* < 0.05). While cells treated with EGF plus LPS significantly increased the T-AOC (Figure 3J,K), CAT (Figure 3A,D), GSH-Px (Figure 3B,E) and SOD (Figure 3C,F) levels compared to cells treated LPS (*p* < 0.05). RT-PCR results showed that there was an increase (*p* < 0.05) in the expression of *CAT* (Figure 3G), *GSH-Px* (Figure 3H) and *SOD2* (Figure 3I) genes in cells treated with EGF, and EGF plus LPS compared to cells treated with LPS.

### 2.4. Oxidative Stress Was Diminished by EGF via Nrf2 Activation

**Figure 4:**
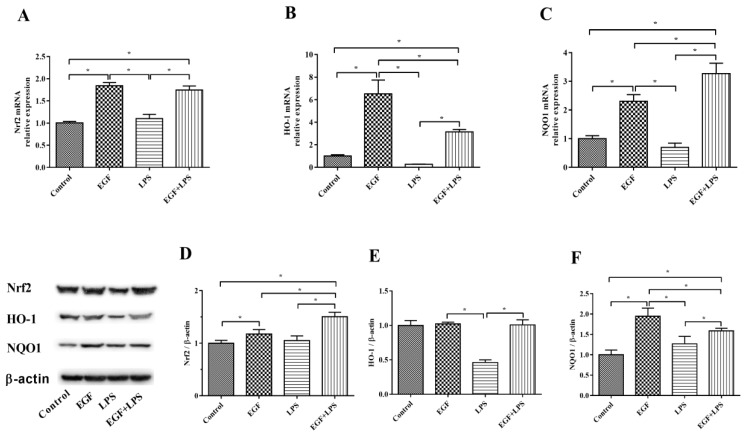
Effects of EGF on Nrf2 signaling pathway-related genes and proteins expression in IPEC-J2 cells challenged by LPS. (**A**) *Nrf2* gene expression; (**B**) *HO-1* gene expression; (**C**) *NQO1* gene expression; (**D**) Nrf2 protein relative abundances; (**E**) HO-1 protein relative abundances; (**F**) NQO1 protein relative abundances. Densitometric values were normalized to β-actin and expressed as a relative level to control values. Data are presented as mean ± SD, *n* = 3, * *p* < 0.05.

The expression of Nrf2-related genes (*Nrf2*, *HO-1* and *NQO1*) and abundances of Nrf2, HO-1 and NQO1 proteins are illustrated in Figure 4. Results showed that cells treated with EGF plus LPS had higher (*p* < 0.05) *Nrf2* (Figure 4A), *HO-1* (Figure 4B) and *NQO1* (Figure 4C) expression than cells treated with LPS. In accordance with above, EGF increased (*p* < 0.05) the protein level of Nrf2 (Figure 4D), HO-1 (Figure 4E), and NQO1 (Figure 4F) when cells were exposed to LPS. These data suggested that EGF would enhance Nrf2 protein expression and upregulate the expression of phase II metabolizing enzymes (such as HO-1 and NQO1) and antioxidative enzymes (SOD, CAT and GSH-Px) to alleviate LPS-induced oxidative injury.

### 2.5. EGF Inhibits LPS-Induced IPEC-J2 Cells Apoptosis

**Figure 5:**
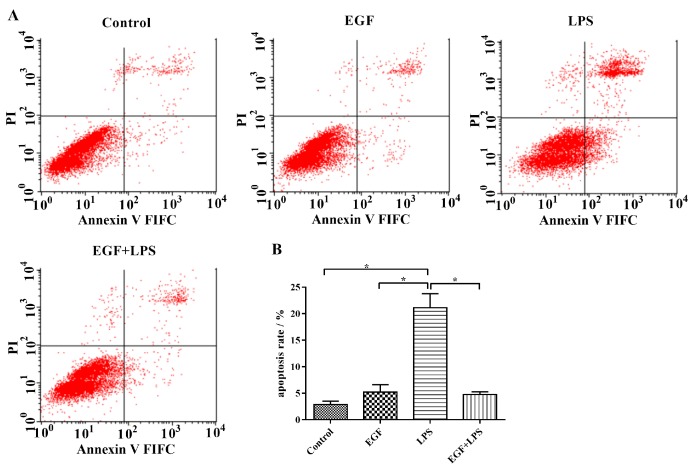
Flow cytometry analyses of EGF against LPS-induced apoptosis in IPEC-J2 cells. (**A**) Representative charts of flow cytometry analyses of apoptosis; (**B**) Flow cytometry analyses of apoptosis using FITC-labeled Annexin V/PI staining, cells situated in the right two quadrants of each plot were regarded as apoptotic cells, data are presented as mean ± SD, *n* = 3, * *p* < 0.05.

According to the result of flow cytometry (Figure 5), the apoptotic percentage in the LPS-treated group (21.13 ± 2.62%) was significantly (*p* < 0.05) higher than that in the Control group (2.88 ± 0.60%), and that in the EGF treated group (5.23 ± 1.39%). On the contrary, treatment of IPEC-J2 cells with EGF showed a significant (*p* < 0.05) inhibition of cell apoptosis induced by LPS (4.77 ± 0.50%). It also indicated that EGF had a protective effect on IPEC-J2 apoptosis.

**Figure 6:**
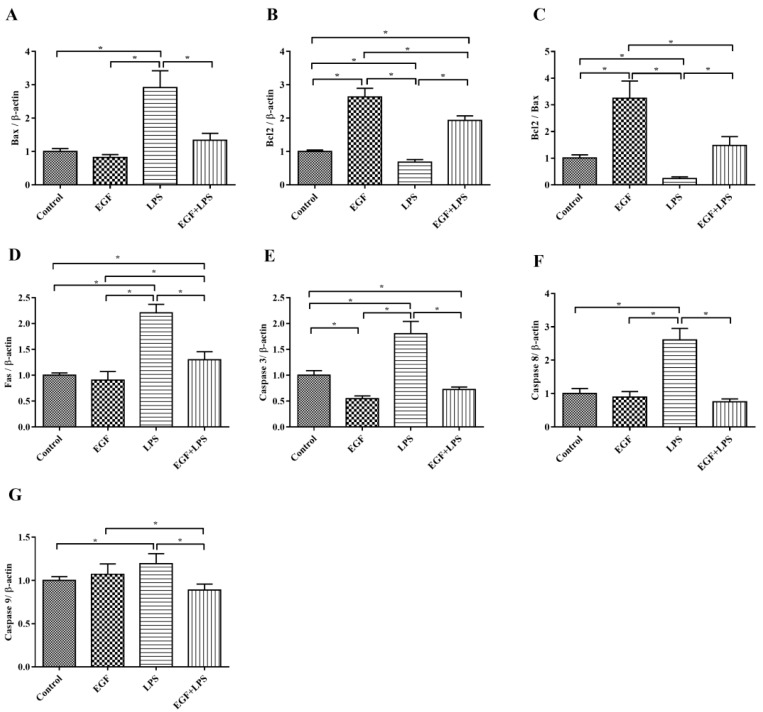
Effects of EGF on pro-apoptotic and anti-apoptotic genes expression induced by LPS. (**A**) *Bax*; (**B**) *Bcl2*; (**C**) *Bcl2*/*Bax*; (**D**) *Fas*; (**E**) *Caspase 3*; (**F**) *Caspase 8*; (**G**) *Caspase 9*. All densitometric values were normalized to *β-actin* and expressed as a relative level to control value. Data are presented as mean ± SD, *n* = 3, * *p* < 0.05.

Next, pro-apoptotic genes (*Fas*, *Bax*, *Cascase 3*, *Cascase 8*, *Cascase 9*) and the anti-apoptotic gene *Bcl2* were examined. The results showed that the expression of pro-apoptotic genes *Bax* (Figure 4A), *Fas* (Figure 6D), *Cascase 3* (Figure 6E), *Cascase 8* (Figure 6F), *Cascase 9* (Figure 6G) were down regulated (*p* < 0.05), and the anti-apoptotic gene *Bcl2* (Figure 6B) was upregulated (*p* < 0.05) when IPEC-J2 cells were treated with EGF plus LPS. Also, the ratio of Bcl2 vs Bax was higher (*p* < 0.05) in the EGF plus LPS group than in the LPS group (Figure 6C). These results indicated that EGF can regulate LPS-induced apoptosis via up-regulating antiapoptotic genes and down-regulating proapoptotic genes.

**Figure 7:**
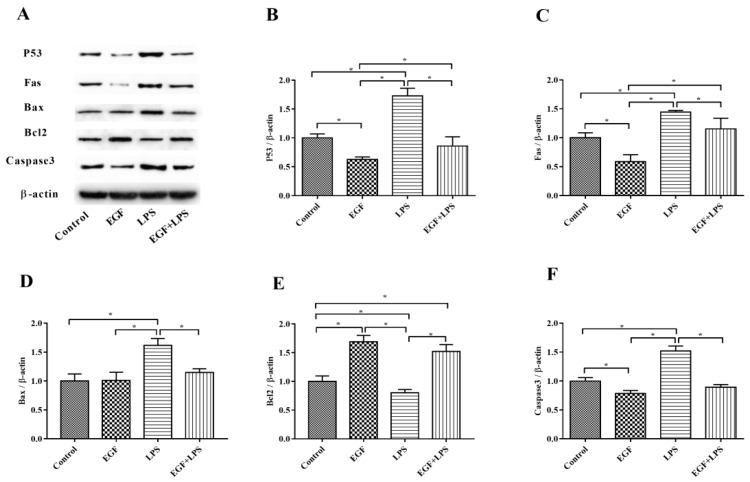
Effects of EGF on pro-apoptotic and anti-apoptotic proteins expression induced by LPS. (**A**) Representative charts of Western blot results; (**B**) P53; (**C**) Fas; (**D**) Bax; (**E**) Bcl2; (**F**) Caspase3. Densitometric values were normalized to β-actin and expressed as a relative level to control values. Data are presented as mean ± SD, *n* = 3, * *p* < 0.05.

Consistently, Western blot analyses also confirmed that EGF can effectively regulate LPS-induced apoptosis. As shown in Figure 7, LPS obviously (*p* < 0.05) caused increased expression of P53, Fas, Bax and Caspase3, and the down-regulation of Bcl2. whereas, EGF significantly (*p* < 0.05) inhibited the expression of P53 (Figure 7B), Fas (Figure 7C), Bax (Figure 7D), Caspase3 (Figure 7F) and promoted the expression of Bcl2 protein (Figure 7E) when cells were challenged by LPS. These results further suggested that EGF can suppress LPS-induced apoptosis in IPEC-J2 cells.

## 3. Discussion

The intestinal mucosa epithelium, formed by a continuous monolayer of proliferating and differentiating IECs, is a fundamental barrier that serves as the body’s first line of defense to provide protection against the outside environment [11]. Intestinal homeostasis is maintained by the dynamic, yet strictly regulated, proliferation and migration of epithelial cells [12]. Recent studies have demonstrated that EGF was able to attenuate the intestinal mucosal epithelial cell injury caused by various stress factors, such as weanling [21,22], alcohol [17], hydrogen peroxide [23], acetaldehyde [15], ischemia/reperfusion [20,24], and NEC [25,26], as well as promote the repair of damaged mucosa epithelium. However, the effect of EGF on oxidative stress and apoptosis in porcine IECs under damage condition is still poorly understood. Hence, we used LPS, a major integral component of the outer membrane of Gram-negative bacteria, which can induce cell injury [11,12,13,14], to establish a cell injury model to investigate the protective effects of EGF on oxidative injury and apoptosis in IPEC-J2 cells. In the present study, we first evaluated the toxicity of EGF and LPS in IPEC-J2 cells; the results showed that EGF at 100 ng/mL can significantly increase cell growth, which is consistent with our previous study, wherein EGF at 100 ng/mL inhibited NaPi-IIb expression [27], and LPS at 1.0 μg/mL caused a dramatic decrease of cell viability. It also showed that cells cultured with EGF for 24 h had higher cell growth rates, and cells cultured with LPS for 24 h had lower cell growth rates. Based on the above results, we used a dose of EGF at 100 ng/mL, a dose of LPS at 1.0 μg/mL, and treated cells for 24 h in subsequent experiments.

Homeostasis of epithelial architecture in the small intestine is regulated by both cell proliferation and apoptosis [3]. However, due to the exposure to various kinds of toxic agents, the intestinal epithelium is often subject to oxidative stress, resulting in enhanced apoptosis [4]. Oxidative stress has been widely implicated in intestinal epithelium apoptosis under both in vivo [7,28] and in vitro conditions [4,29]. Oxidative stress in cultured cells has been assessed by markers such as LDH release [4,30] and MDA production [31]. The present study showed that the levels of LDH and MDA increased after treatment with LPS, which demonstrated that IPEC-J2 suffered oxidative stress. EGF can reduce IPEC-J2 apoptosis induced by oxidative stress, which was confirmed by the improved cell viability and decreased apoptosis rate in this study.

Apoptosis may be activated by the intrinsic (mitochondria apoptosis pathway) or by the extrinsic pathway (death receptor pathway) [32]. The extrinsic apoptosis pathway also known as death receptor pathway involves direct interaction with a death receptor, such as Fas, which enables the catalytic activity of caspase-8 [3,33]. The intrinsic apoptosis is a mitochondria-dependent pathway, which is characterized by the activation of one or more pro-apoptotic members of the Bcl-2 family of proteins, which act as the gate keepers of cell death in the mitochondria [28,30]. This family contains both proapoptotic (Bax, Bak, Bad) and antiapoptotic (Bcl-2, Bcl-XL) proteins; wherein the family of proteins that control the intrinsic pathway is known as Bcl-2. Bcl-2 is an important cellular component which can protect against apoptotic cell death, while Bax proteins have been confirmed to promote apoptosis [32]. The balance between Bcl-2 and Bax is responsible for the determination of either cell death or cell recovery [3]. Bcl-2 down-expression leads to the release of cytochrome c from the damaged mitochondria, then, by interacting with apoptotic protease activating factor 1 (APAF-1) it enables the catalytic activity of caspase-9 [3,32]. The activation of extrinsic (mediated by caspase-8) and intrinsic (mediated by caspase-9) pathways leads to activation of caspase-3, resulting in the morphological and biochemical changes associated with apoptosis [34]. In the present study, the increased pro-apoptotic genes, such as *Fas*, *Bax*, *caspase-3*, *caspase-8* and *caspase-9* expressions and the decreased of anti-apoptotic gene *Bcl-2* expressions in IPEC-J2 cells challenged by LPS suggest that both mitochondria-dependent apoptosis and Fas-dependent apoptosis are involved in IPEC-J2 cells. The gene expression of *Bcl-2*, *Fas*, *Bax*, *caspase-3*, *caspase-8* and *caspase-9* were reversed by EGF in IPEC-J2 cells after exposure to LPS. Consistently, Western blot analyses also confirmed that EGF can effectively regulate LPS-induced apoptosis. EGF significantly inhibited the expression of, Fas, Bax, Caspase3 and promoted the expression of Bcl2 proteins when cells were challenged by LPS. It could be concluded that EGF, through inhibiting Fas-dependent pathway as well as promoting Bcl-2 expression to attenuate IPEC-J2 cells apoptosis induced by LPS. P53 is one of the most extensively characterized tumor suppressor proteins, which can regulate apoptosis [35]. P53 can promote pro-apoptotic proteins (such as Bax, Bid, PUMA, and Noxa) and inhibit Bcl-2 expression, which induce a caspase cascade to trigger apoptosis [36]. In the current study, P53 protein was inhibited in cells treated with EGF plus LPS compared to those treated with LPS. This indicated that EGF can regulate apoptosis in IPEC-J2 cells challenged by LPS through inhibiting P53 protein expression.

The nuclear factor erythroid 2-related factor 2 (Nrf2) plays a vital role in maintaining cellular homeostasis, when exposure of cells to chemical or oxidative stress, through its ability to regulate a wide array of genes related to detoxification and antioxidant function [37]. Kelch-like ECH-associated protein-1 (Keap1) is a repressor of Nrf2 activity and functions as an adaptor for Cul3/Rbx1 E3 ubiquitin ligase-mediated degradation [38]. Under resting conditions, Nrf2 is retained in the cytosol, bound to Keap1, and is marked for ubiquitin-dependent proteasomal degradation [39]. Upon oxidative stress, Nrf2 parts from Keap1 and translocates into the nucleus where it binds to the antioxidant response elements (AREs) of target genes including phase II metabolizing enzymes, such as heme oxygenase (HO-1) and quinone oxidoreductase (NQO1), and antioxidant enzymes such as SOD, CAT and GSH-Px [39,40,41]. HO-1 is the rate-limiting enzyme that catalyzes haem to biliverdin, carbon monoxide (CO) and free iron [42]. HO-1 and its metabolic product (i.e., CO and bilirubin) have antioxidative and anti-inflammatory properties [43]; it is a known Nrf2 target gene induced in an Nrf2-dependent manner [39,44]. NQO1 is another Nrf2-meadiated phase II metabolizing enzyme, which through reducing ROS production and NADPH oxidase enzyme activity alleviates oxidative injury [45]. Antioxidant enzymes such as SOD, CAT and GSH-Px play important roles in maintaining the redox homeostasis in cells [46]. In the current study, we observed that EGF can activate Nrf2, when IPEC-J2 cells were stimulated by LPS, and led to the activation of phase II metabolizing enzymes (HO-1, NQO1) and antioxidant enzymes (SOD, CAT, GSH-Px). It demonstrated that EGF would enhance Nrf2 protein expression and upregulate the expression of phase II metabolizing enzymes (such as HO-1 and NQO1) and anti-oxidative enzymes (SOD, CAT and GSH-Px) to reduce LPS-induced oxidative injury in IPEC-J2 cells.

Evidence has proven that both mitochondria-dependent apoptosis and Fas-dependent apoptosis are essential for oxidative stress-induced apoptosis in epithelial cells [28,29,47]. Bcl2, an anti-apoptotic protein localized to mitochondria, has been shown to inhibit cytochrome c release and protect against oxidative stress-induced apoptosis [47]. Furthermore, evidence also reported that ROS scavengers, such as GSH and SOD, can inhibit Fas-induced apoptosis in different cell types [28,48,49]. In the present study, we found that an increase in Fas, Bax, caspase-9, caspase-8 and caspase-3 expressions coincided with decreases in the CAT, GSH-Px and SOD activities in the LPS-treated IPEC-J2 cells, and these were reversed by EGF. These results suggest that the ability of EGF inhibit oxidative stress-induced apoptosis is associated with both Fas-dependent pathway and mitochondria-dependent pathway.

**Figure 8:**
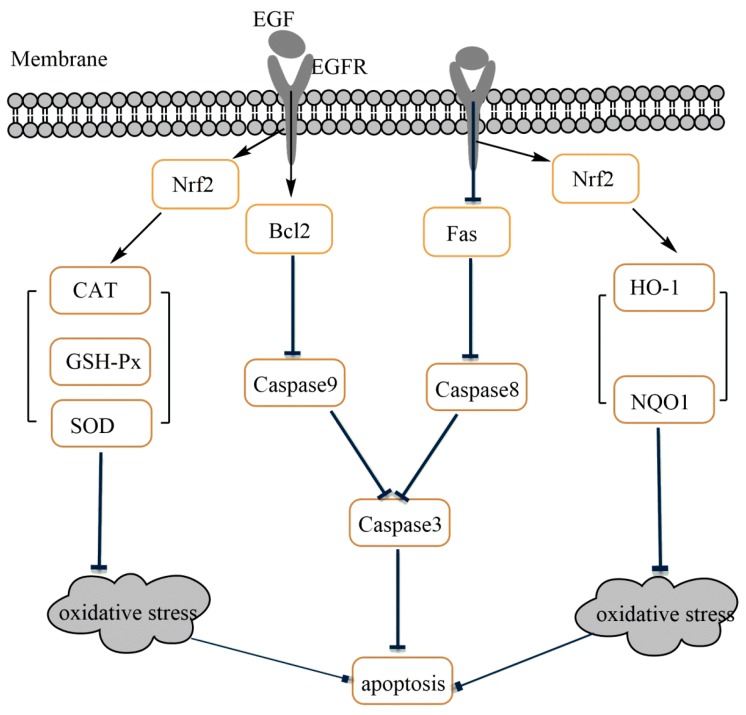
Possible mechanism of EGF on the regulation of oxidative stress-induced apoptosis in IPEC-J2 cells.

In summary, the present study shows that EGF exhibited potent protective effects on IPEC-J2 cells against LPS-induced cell damage, through enhanced Nrf2-mediated phase II metabolizing enzymes (HO-1 and NQO1) and antioxidative enzymes (SOD, CAT and GSH-Px) expression to alleviate oxidative injury, and then protected IPEC-J2 cells from apoptosis induced by LPS (Figure 8).

## 4. Materials and Methods

### 4.1. Regents and Antibodies

EGF was purchased from Peprotech (Rocky Hill, NJ, USA). LPS was purchased from Sigma-Aldrich (Saint Louis, MO, USA). Fetal bovine serum (FBS), Trypsin/EDTA and antibiotics (Penicillin-Streptomycin for Cell Culture) were from GIBCO (Carlsbad, CA, USA). Dulbecco’s modified Eagle’s F12 Ham medium (HyClone^TM^ DMEM/F12 1:1 media) was purchased from GE Healthcare life sciences (South Logan, UT, USA). Plastic culture plates were manufactured by Corning Inc. (Corning, NY, USA). CCK-8 Assay Kit, BCA protein assay reagent, LDH Assay Kit, T-AOC Assay Kit, CAT Assay Kit, GSH-Px Assay Kit, SOD Assay Kit, and MDA Assay Kit were purchased from Nanjing Jiancheng Bioengineering Institute (Nanjing, China). PBS, RIPA Lysis Buffer R2220 were purchased from Solarbio (Beijing, China). TRIzol Reagent was obtained from Invitrogen (Carlsbad, CA, USA). Annexin V-FITC/PI kits was obtained from Keygen Biotech (Nanjing, China).The primary antibodies against Nrf2, HO-1, NQO1, P53, Bax, Bcl2, Caspase3, β-actin, and the secondary antibody Goat Anti-Rabbit IgG/HRP used in Western blot analyses were all purchased from Proteintech (Rosemont, IL, USA). The primary antibody against Fas was purchased from Abcam (Cambridge, MA, USA).

### 4.2. Cell Culture

Porcine intestinal epithelial cells (IPEC-J2) were kindly provided by Dr. Bie Tan (Institute of Subtropical Agriculture, Chinese Academy of Science, Changsha, China). IPEC-J2 cells were cultured in DMEM/F12 medium containing 10% FBS, 1% antibiotics (Penicillin-Streptomycin), and grown in a humidified incubator at 37 °C with 5% CO_2_ and 95% air.

### 4.3. Evaluation of Toxicity of EGF and LPS in IPEC-J2 Cells

Cell numbers was evaluated using a CCK-8 assay. IPEC-J2 cells were seeded in 96-well plates (1 × 10^4^/well) and cultured in DMEM/F12 with 10% FBS and 1% antibiotics for 24 h. Cells were treated with EGF at concentrations of 0, 50, 100, 150, 200, 250 ng/mL, or treated with LPS at concentrations of 0, 0.1, 1.0, 10 μg/mL for 24 h, then added 10 mL CCK-8 reagent, further cultured for 3 h, then measured the optical density (OD) at a wavelength of 450 nm used an enzyme-linked immune detector (Bio-Rad, Hercules, CA, USA). Cell numbers are presented as the percent of control cells. Experiments were performed in 6 times.

### 4.4. Evaluation of Time-Dependent Effect of EGF and LPS in IPEC-J2 Cells

IPEC-J2 cells were seeded in 96-well plates (1 × 10^4^/well) and cultured in DMEM/F12 with 10% FBS and 1% antibiotics for 24 h. Cells were treated with EGF at concentrations of 100 ng/mL, or treated with LPS at concentrations of 1.0 μg/mL (determined by toxicity experiment) for 6, 12, 24, 36, 48 h, then added 10 mL CCK-8 reagent respectively, further cultured for 3 h, then measured the OD at a wavelength of 450 nm used an enzyme-linked immune detector (Bio-Rad, Hercules, CA, USA). Cell numbers are presented as the percent of control cells. Experiments were performed in 6 times.

### 4.5. Effect of EGF on Cell Growth Challenged by LPS

IPEC-J2 cells were seeded in 96-well plates (1 × 10^4^/well) and cultured in DMEM/F12 with 10% FBS and 1% antibiotics for 24 h, then IPEC-J2 cells were treated for 24 h (determined by time-dependent experiment) with: (1) EGF (0 ng/mL) + LPS (0 μg/mL) (Control group); (2) EGF (100 ng/mL) + LPS (0 μg/mL) (EGF group); (3) EGF (0 ng/mL) + LPS (1 μg/mL) (LPS group); and (4) EGF (100 ng/mL) + LPS (1 μg/mL) (EGF + LPS group), then added 10 mL CCK-8 reagent respectively, further cultured for 3 h, and measured the OD at a wavelength of 450 nm used an enzyme-linked immune detector (Bio-Rad, Hercules, CA, USA). Cell numbers are presented as the percent of control cells. Experiments were performed in 6 times.

### 4.6. Determination of LDH and MDA Release

IPEC-J2 cells were seeded in 6-well plates (1 × 10^5^/well) and cultured in DMEM/F12 with 10% FBS and 1% antibiotics for 24 h, then IPEC-J2 cells were treated for 24 h with: (1) EGF (0 ng/mL) + LPS (0 μg/mL) (Control group); (2) EGF (100 ng/mL) + LPS (0 μg/mL) (EGF group); (3) EGF (0 ng/mL) + LPS (1 μg/mL) (LPS group); and (4) EGF (100 ng/mL) + LPS (1 μg/mL) (EGF + LPS group). After a 24 h of incubation, 1.0 mL cell supernatant was collected and stored at −20 °C until analysis. Cells in 6-well culture plates were gently washed with PBS for twice, then RIPA Lysis Buffer R2220 (containing 1% PMSF) was used to lyse IPEC-J2 cells according to the instructions of the manufacturer. Cellular protein was determined using the bicinchoninic acid (BCA) protein assay reagent. The determination of LDH levels used LDH assay kit (A020-2) from Nanjing Jiangcheng Biotechnology Institute as previously reported [50]. The determination of MDA levels used MDA assay kit (A003-2) from Nanjing Jiangcheng Biotechnology Institute as previously reported [51]. Experiments were performed in 6 times.

### 4.7. Flow Cytometry

IPEC-J2 cells were seeded in 6-well plates (1 × 10^5^/well) and cultured in DMEM/F12 with 10% FBS and 1% antibiotics for 24 h, then IPEC-J2 cells were treated for 24 h with: (1) EGF (0 ng/mL) + LPS (0 μg/mL) (Control group); (2) EGF (100 ng/mL) + LPS (0 μg/mL) (EGF group); (3) EGF (0 ng/mL) + LPS (1 μg/mL) (LPS group); and (4) EGF (100 ng/mL) + LPS (1 μg/mL) (EGF + LPS group). After a 24 h of incubation, collected cells were costained with 10 μM Annexin V-FITC and propidium iodide (PI) (Annexin V-FITC/PI kits) for 15 min at room temperature in the dark. Apoptotic cells were identifed using a BD FACSCalibur flow cytometer (BD Biosciences, San Diego, CA, USA). The data were analyzed using the sofware CELLQuest. Experiments were performed in triplicate.

### 4.8. Determination of T-AOC, CAT, GSH-Px, SOD Levels

IPEC-J2 cells were seeded in 6-well plates (1 × 10^5^/well) and cultured in DMEM/F12 with 10% FBS and 1% antibiotics (Penicillin-Streptomycin) for 24 h, then IPEC-J2 cells were treated for 24 h with: (1) EGF (0 ng/mL) + LPS (0 μg/mL) (Control group); (2) EGF (100 ng/mL) + LPS (0 μg/mL) (EGF group); (3) EGF (0 ng/mL) + LPS (1 μg/mL) (LPS group); and (4) EGF (100 ng/mL) + LPS (1 μg/mL) (EGF + LPS group). After a 24 h of incubation, 1.0 mL cell supernatant was collected and stored at −20 °C until analysis. Cells in 6-well culture plates were gently washed with PBS for twice, then RIPA Lysis Buffer R2220 (containing 1% PMSF) was used to lyse IPEC-J2 cells according to the instructions of the manufacturer. Cellular protein was determined using the bicinchoninic acid (BCA) protein assay reagent at 562 nm according to the instructions of the manufacturer. The extracted protein sample stored at −20 °C until analysis. The determination of T-AOC, CAT, GSH-Px, SOD levels in cells and cell supernatants used T-AOC (A015-1), CAT (A007-1-1), GSH-Px (A005), SOD (A001-3) assay kit respectively from Nanjing Jiang cheng Biotechnology Institute as previously reported [51]. Experiments were performed in triplicate.

### 4.9. Real-Time PCR (RT-PCR) Analysis of Gene Expression

After a 24 h of EGF and/or LPS treatment, total cell RNA was extracted and purified using TRIzol Reagent (Invitrogen, Carlsbad, CA, USA) following the protocol provided by the manufacturer. Real-time PCR was performed as described previously [52]. The primers of genes (Sangon Biotech, Shanghai, China) were shown in Table 1. *β-actin* was used as a housekeeping gene to normalize target gene transcript levels. Relative expression was normalized and expressed as a ratio to the expression in control group.

### 4.10. Western Blot Analysis

After a 24 h of EGF and/or LPS treatment, cells in 6-well culture plates were gently washed with PBS for twice, then RIPA Lysis Buffer R2220 (containing 1% PMSF) was used to lyse IPEC-J2 cells according to the instructions of the manufacturer. Cellular protein concentration was determined using the bicinchoninic acid (BCA) protein assay reagent at 562 nm according to the instructions of the manufacturer. Equal amount of protein samples of cell lysate was loaded for SDS-PAGE and subsequently transferred to PVDF membrane. The membrane was blocked with PBST buffer containing 5% skim-milk for 1 h at room temperature followed by overnight hybridization at 4 °C with the indicated primary anti-bodies: anti-Nrf2, anti-HO-1, anti-NQO1, anti-P53, anti-Fas, anti-Bax, anti-Bcl2, anti-Caspase3 and anti-β-actin. After incubation with secondary antibody (HRP goat anti-rabbit IgG) for 1 h, signals were detected using enhanced chemiluminescence kits (ECL-Plus, Thermo, Waltham, MA, USA), and then scanned for detection of fluorescence using the BioRad gel detection system. All densitometric values were normalized to β-actin and expressed as a relative level to control value. Experiments were performed in triplicate.

### 4.11. Statistical Analysis

All data are expressed as mean ± standard deviation (SD). Data are performed by one-way ANOVA procedure of IBM SPSS statistics 21.0 (SPSS, Inc., Chicago, IL, USA). Difference of means of two groups was determined by the Student’s Paired *t* test. *p* < 0.05 were taken to indicate statistical significance.

## Figures and Tables

**Table 1:** Primers Used for Quantitative Reverse Transcription PCR.

Gene	Primers Squence	Product Length
*β-actin*	F: 5’-CATCCTGCGTCTGGACCTGG-3′ R: 5’-TAATGTCACGCACGATTTCC-3′	116 bp
*Fas*	F: 5’-GACCCAGAATACCAAGTGCAG-3′ R: 5’-ATGTCTTCAAGTCCACACGAG-3′	99 bp
*Bax*	F: 5’-GGTCGCGCTTTTCTACTTTGCC-3′ R: 5’-TCCAGCCCATGATGGTCCT-3′	89 bp
*Bcl2*	F: 5’-CCAAGATCATTCTGACGGAGT-3′ R: 5’-GGTATCATAAGCCAGCAACGAA-3′	228 bp
*Caspase 3*	F: 5’-CACCCGGTTACTATTCCTG-3′ R: 5’-GCATTGACACAATACACGG-3′	207 bp
*Caspase 8*	F: 5’-AGCCTGCTTGATATTTTCGT-3′ R: 5’-GATCCTTCCCAGCAAGCTC-3′	114 bp
*Caspase 9*	F: 5’-TCCCATACCAGGAAGGCCCAA-3′ R: 5’-TCGATGTACCAGGAACCGCTCT-3′	145 bp
*SOD2*	F: 5’-ACAC CGAGTACATCAAGCTCT-3′ R: 5’-AAATACCTGAACAAGCCGCATT-3′	113 bp
*GPXs*	F: 5’-CGGACCACCTGTTGAAAGCTC-3′ R: 5’-TCCGCCAGTTCTTGTTGTCCA-3′	127 bp
*CAT*	F: 5’-CCACTAATGTCCAGCGTCT-3′ R: 5’-CAGCCTTATTCACCACTACCTG-3′	159 bp
*Nrf2*	F: 5’-AGTGCAAGGCGGAGGTGA-3′ R: 5’-AGCCCGTTGGTGAACATAG-3′	235 bp
*HO-1*	F: 5’-TTGTGTCTCGTGTTTCCGTCT-3′ R: 5’-CCCCTCACCCCACCTTGCT-3′	112 bp
*NQO1*	F: 5’-GATCATACTGGCCCACTCCG-3′ R: 5’-GAGCAGTCTCGGCAGGATAC-3′	200 bp
